# ﻿Three new species of *Diploderma* Hallowell, 1861 (Squamata, Agamidae) from the Hengduan Mountain Region, south-western China

**DOI:** 10.3897/zookeys.1131.86644

**Published:** 2022-11-22

**Authors:** Shuo Liu, Mian Hou, Dingqi Rao, Natalia B. Ananjeva

**Affiliations:** 1 Kunming Natural History Museum of Zoology, Kunming Institute of Zoology, Chinese Academy of Sciences, Kunming, Yunnan 650223, China Kunming Institute of Zoology, Chinese Academy of Sciences Kunming China; 2 Kunming Institute of Zoology, Chinese Academy of Sciences, Kunming, Yunnan 650201, China Kunming Natural History Museum of Zoology Kunming China; 3 College of Continuing (Online) Education, Sichuan Normal University, Chengdu, Sichuan 610066, China Sichuan Normal University Chengdu China; 4 Zoological Institute, Russian Academy of Sciences, Universitetskaya nab., 1, St. Petersburg 199034, Russia Zoological Institute, Russian Academy of Sciences St. Petersburg Russia

**Keywords:** Molecular, morphological, ND2, Sichuan, taxonomy, Yunnan

## Abstract

Three new species of *Diploderma* are described from the Hengduan Mountain Region in south-western China, based on morphological and genetic data. The first new species from Yulong County, Yunnan Province is morphologically most similar and phylogenetically closely related to *D.brevicauda*, but it can be diagnosed from the latter by having a relatively longer tail; the second new species from Xiangcheng County, Sichuan Province is phylogenetically closely related to *D.bowoense*, but it can be diagnosed from the latter by the absence of a distinct gular spot; and the third new species from Yongsheng County, Yunnan Province is phylogenetically closely related to *D.yulongense*, but it can be diagnosed from the latter by having different colourations of the ventral and ventrolateral surfaces of the body. Taxonomy and diversity survey are the basis of species conservation, our discoveries contributing to better conservation of the species of this genus.

## ﻿Introduction

*Diploderma* Hallowell, 1861, is a genus including 36 species recognised currently (Uetz et al. 2022; Wang et al. 2022). Of the total diversity, 34 species are distributed in China, of which 22 species are only distributed in the Hengduan Mountain Region of south-western China (Wang et al. 2021a, 2022).

In the Hengduan Mountain Region, species of *Diploderma* mainly inhabit the hot-dry river valleys and most species are micro-endemic and only found in a specific section of a given river valley (Wang et al. 2022). Amongst the river valleys in the Hengduan Mountain Region, the Jinsha River Valley has the highest diversity of this genus, especially the upper and middle Jinsha River Valley (Wang et al. 2021a, b).

**Figure 1.**
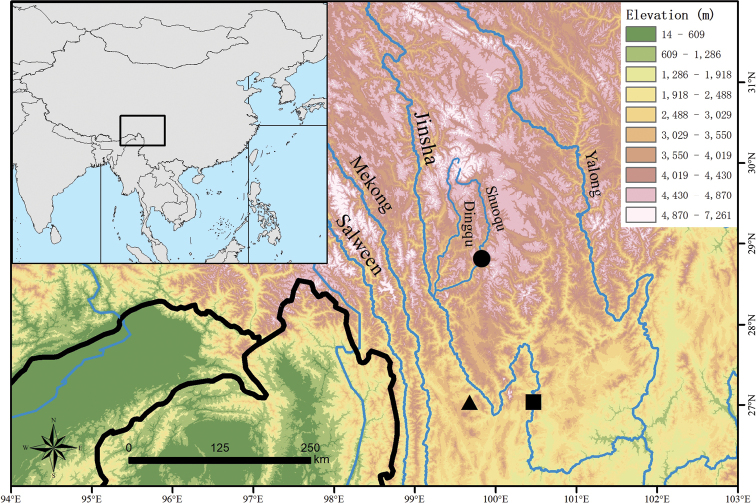
Map showing the type localities of *Diplodermalimingense* sp. nov. (black triangle), *Diplodermashuoquense* sp. nov. (black dot) and *Diplodermayongshengense* sp. nov. (black square) in the Hengduan Mountain Region, south-western China. The elevation data were obtained from Geospatial Data Cloud (2022).

During our field survey in the Hengduan Mountain Region, China, in April 2022, some specimens of *Diploderma* were collected from the middle Jinsha River Valley in Yongsheng County, the area nearby the upper Jinsha River in Yulong County and the valley of a tributary of the upper Jinsha River in Xiangcheng County in Yunnan and Sichuan provinces, respectively (Fig. 1). Morphologically, these specimens could not be assigned to any recognised species of the genus. Phylogenetic analysis indicated that these populations represent three distinct, undescribed lineages. Herein, we describe these populations as three new species of *Diploderma*.

## ﻿Materials and methods

### ﻿Sampling

Specimens were all collected during the day. Photographs were taken to document the colour pattern in life prior to euthanasia. Liver tissues were stored in 99% ethanol and lizards were preserved in 75% ethanol. Specimens were deposited at Kunming Natural History Museum of Zoology, Kunming Institute of Zoology, Chinese Academy of Sciences (**KIZ**).

### ﻿Morphology

Specimens were measured using a digital caliper to the nearest 0.1 mm. Measurements were taken on the left side of the specimen and values for paired pholidosis characters were recorded on both sides of the body, with counts provided in left/right order. The following morphometric characters were measured following Wang et al. (2022):

**F4S** fourth finger subdigital lamellae number, subdigital lamellae scale from the base between third and fourth finger to the tip of fourth finger, excluding the claw;

**FLL** fore-limb length, measured between the point of insertion at axillary to the tip of fourth finger, excluding the claw, measured as the straightened limb;

**HD** head depth, measured as the perpendicular distance at the temporal region of head;

**HL** head length, measured from the tip of snout to the rear border of the angle of jaw;

**HLL** hind-limb length, measured between the point of insertion at groin to the tip of fourth toe, excluding the claw, measured as the straightened limb;

**HW** head width, measured between the widest points of the head;

**IL** infralabial scale number, enlarged, modified labial scales from mental to the corner of mouth;

**MD** mid-dorsal crest scale number, modified crest scales longitudinally from the first nuchal crest to the scale above cloaca;

**NSL** nasal-supralabials scale rows, number of horizontal rows of small scales between the first supralabial and the nasal;

**SEL** snout-eye length, measured between the tip of snout and anterior edge of orbital bone;

**SL** supralabial scale number, enlarged, modified labial scales from rostral to the corner of mouth;

**SOR** suborbital scale rows, longitudinal rows of scales between supralabials and inferior-most edge of orbit circle, excluding fine ciliary scales in the orbit;

**SVL** snout-vent length, measured from the snout tip to anterior edge of the cloaca;

**T4L** fourth toe length, measured between the tip of fourth toe to the base between third and fourth toe, excluding the claw;

**T4S** fourth toe subdigital lamellae number, subdigital lamellae scales from the base between third and fourth toe to the tip of fourth toe, excluding the claw;

**TAL** tail length, measured from the anterior edge of the cloaca to the tip of tail;

**TRL** trunk length, measured between the limb insertion points between axillary and groin;

**VN** ventral scale number, ventral body scales counted in a straight line along the medial axis between the transverse gular fold and the anterior edge of cloaca.

We compared morphological characters of the new species with other members of the genus relying on original species descriptions (Hallowell 1861; Günther 1864; Anderson 1878; Boulenger 1906, 1918; Barbour and Dunn 1919; Stejneger 1924; Mertens 1926; Smith 1935; Gressitt 1936; Bourret 1937; Song 1987; Ota 1989; Ota et al. 1998; Li et al. 2001; Gao and Hou 2002; Manthey et al. 2012; Wang et al. 2015, 2016, 2017, 2019b, d, 2021a, b, 2022; Ananjeva et al. 2017; Rao et al. 2017; Liu et al. 2020) and the additional data from Wu et al. (2005), Manthey (2008) and Wang et al. (2017, 2018, 2019b, c, 2021a).

### ﻿Molecular analysis

Total genomic DNA for the new collected specimens was extracted from liver tissues with the standard extraction method (Sambrook et al. 1989). The mitochondrial gene NADH dehydrogenase subunit 2 (ND2) was amplified and sequenced by using published primers (Wang et al. 2019a). PCR and sequencing methods followed Liu et al. (2020). Sequences were edited and manually managed using SeqMan in Lasergene 7.1 (DNASTAR Inc., Madison, WI, USA) and MEGA 11 (Tamura et al. 2021). Representative species of *Pseudocalotes* Fitzinger were chosen as outgroups according to Wang et al. (2022). Genetic data for 32 species of *Diploderma* and two species of outgroup taxa were obtained from GenBank (Table 1).

**Table 1.** GenBank accession numbers for the sequences used in this study.

Species	Voucher	Locality	Accession Numbers
*Diplodermaangustelinea*	KIZ 029704	Muli, Sichuan, China	MT577930
*Diplodermaangustelinea*	KIZ 029705	Muli, Sichuan, China	MT577924
*Diplodermaaorun*	KIZ 032733	Benzilan, Yunnan, China	MT577938
*Diplodermaaorun*	KIZ 032734	Benzilan, Yunnan, China	MT577939
*Diplodermabatangense*	KIZ 09404	Zhubalong, Tibet, China	MK001412
*Diplodermabatangense*	KIZ 019276	Batang, Sichuan, China	MK001413
*Diplodermabrevicauda*	KIZ 044304	Lijiang, Yunnan, China	MW506023
*Diplodermabrevicauda*	KIZ 044305	Lijiang, Yunnan, China	MW506021
*Diplodermabrevicauda*	KIZ 044306	Lijiang, Yunnan, China	MW506022
*Diplodermabowoense*	KIZ 044757	Muli, Sichuan, China	MW506020
*Diplodermabowoense*	KIZ 044758	Muli, Sichuan, China	MW506019
*Diplodermabrevipes*	NMNS 19607	Taiwan, China	MK001429
*Diplodermabrevipes*	NMNS 19608	Taiwan, China	MK001430
*Diplodermachapaense*	KIZ 034923	Lvchun, Yunnan, China	MG214263
*Diplodermachapaense*	ZMMU NAP-01911	Chapa, Vietnam	MG214262
*Diplodermadrukdaypo*	KIZ 027627	Jinduo, Tibet, China	MT577950
*Diplodermadrukdaypo*	KIZ 027628	Zhuka, Tibet, China	MT577952
*Diplodermadymondi*	KIZ 040639	Dongchuan, Yunnan, China	MK001422
*Diplodermadymondi*	KIZ 040640	Dongchuan, Yunnan, China	MK001423
*Diplodermaflaviceps*	KIZ 01851	Luding, Sichuan, China	MK001416
*Diplodermaflaviceps*	KIZ 01852	Luding, Sichuan, China	MK001417
*Diplodermaflavilabre*	KIZ 032692	Baiyu,Sichuan, China	MT577916
*Diplodermaflavilabre*	KIZ 032694	Baiyu,Sichuan, China	MT577917
*Diplodermaformosgulae*	KIZ 044420	Deqin, Yunnan, China	MW506024
*Diplodermaformosgulae*	KIZ 044421	Deqin, Yunnan, China	MW506025
*Diplodermaiadinum*	KIZ 027697	Yunling, Yunnan, China	MT577956
*Diplodermaiadinum*	KIZ 027702	Yunling, Yunnan, China	MT577957
*Diplodermalaeviventre*	KIZ 014037	Basu, Tibet, China	MK001407
*Diplodermalaeviventre*	KIZ 027691	Basu, Tibet, China	MT577892
*Diplodermaluei*	NMNS 19604	Taiwan, China	MK001433
*Diplodermaluei*	NMNS 19605	Taiwan, China	MK001434
*Diplodermamakii*	NMNS 19609	Taiwan, China	MK001431
*Diplodermamakii*	NMNH 19610	Taiwan, China	MK001432
*Diplodermamenghaiense*	KIZ L0030	Menghai, Yunnan, China	MT598655
*Diplodermamenghaiense*	KIZ L0031	Menghai, Yunnan, China	MT598656
*Diplodermamicangshanense*	KIZ 032801	Shiyan, Hubei, China	MK578665
*Diplodermamicangshanense*	KIZ 023231	Xixia, Henan, China	MK578664
*Diplodermapanchi*	KIZ 032715	Yajiang, Sichuan, China	MT577946
*Diplodermapanchi*	KIZ 032716	Yajiang, Sichuan, China	MT577944
*Diplodermapanlong*	KIZ 040137	Miansha, Sichuan, China	MT577906
*Diplodermapanlong*	KIZ 040138	Miansha, Sichuan, China	MT577907
*Diplodermapolygonatum*	NMNS 19598	Taiwan, China	MK001427
*Diplodermapolygonatum*	NMNS 19599	Taiwan, China	MK001428
*Diplodermaqilin*	KIZ 028332	Balong, Yunnan, China	MT577941
*Diplodermaqilin*	KIZ 028333	Balong, Yunnan, China	MT577942
*Diplodermaqilin*	KIZ 028335	Balong, Yunnan, China	MT577943
*Diplodermaslowinskii*	CAS 214906	Gongshan, Yunnan, China	MK001405
*Diplodermaslowinskii*	CAS 214954	Gongshan, Yunnan, China	MK001406
*Diplodermasplendidum*	KIZ 015973	Yichang, Hubei, China	MK001418
*Diplodermasplendidum*	LSUMZ 81212	Unknown	AF288230
*Diplodermaswild*	KIZ 034914	Panzhihua, Sichuan, China	MN266299
*Diplodermaswild*	KIZ 034894	Panzhihua, Sichuan, China	MN266300
*Diplodermaswinhonis*	NMNS 19592	Taiwan, China	MK001419
*Diplodermaswinhonis*	NMNS 19593	Taiwan, China	MK001420
*Diplodermavarcoae*	WK-JK 011	Yuxi, Yunnan, China	MT577903
*Diplodermavarcoae*	KIZ 026132	Mengzi, Yunnan, China	MK001421
*Diplodermavela*	KIZ 019299	Quzika, Tibet, China	MK001414
*Diplodermavela*	KIZ 034925	Quzika, Tibet, China	MK001415
*Diplodermayangi*	SWFU 005410	Chayu, Tibet, China	OL449603
*Diplodermayangi*	SWFU 005412	Chayu, Tibet, China	OL449604
*Diplodermayulongense*	KIZ 028291	Hutiaoxia, Yunnan, China	MT577921
*Diplodermayulongense*	KIZ 028292	Hutiaoxia, Yunnan, China	MT577922
*Diplodermayulongense*	KIZ 028300	Baishuitai, Yunnan, China	MT577923
*Diplodermayulongense*	KIZ 09399	Xianggelila, Yunnan, China	MK001410
*Diplodermayulongense*	KIZ 043196	Xianggelila, Yunnan, China	MK001411
*Diplodermayunnanense*	CAS 242271	Baoshan, Yunnan, China	MK001408
*Diplodermayunnanense*	KIZ 040193	Yingjiang, Yunnan, China	MK578658
*Diplodermazhaoermii*	KIZ 019564	Wenchuan, Sichuan, China	MK001425
*Diplodermazhaoermii*	KIZ 019565	Wenchuan, Sichuan, China	MK001426
*Diplodermalimingense* sp. nov.	KIZ2022013	Liming, Yunnan, China	OP428781
*Diplodermalimingense* sp. nov.	KIZ2022014	Liming, Yunnan, China	OP428782
*Diplodermalimingense* sp. nov.	KIZ2022015	Liming, Yunnan, China	OP428783
*Diplodermalimingense* sp. nov.	KIZ2022017	Liming, Yunnan, China	OP428784
*Diplodermashuoquense* sp. nov.	KIZ2022004	Xiangcheng, Sichuan, China	OP428773
*Diplodermashuoquense* sp. nov.	KIZ2022005	Xiangcheng, Sichuan, China	OP428774
*Diplodermashuoquense* sp. nov.	KIZ2022006	Xiangcheng, Sichuan, China	OP428775
*Diplodermashuoquense* sp. nov.	KIZ2022007	Xiangcheng, Sichuan, China	OP428776
*Diplodermayongshengense* sp. nov.	KIZ2022008	Yongsheng, Yunnan, China	OP428777
*Diplodermayongshengense* sp. nov.	KIZ2022009	Yongsheng, Yunnan, China	OP428778
*Diplodermayongshengense* sp. nov.	KIZ2022010	Yongsheng, Yunnan, China	OP428779
*Diplodermayongshengense* sp. nov.	KIZ2022011	Yongsheng, Yunnan, China	OP428780
*Pseudocalotesbrevipes*	MVZ 224106	Vinh Phuc, Vietnam	AF128502
*Pseudocaloteskakhiensis*	KIZ 015975	Gongshan, Yunnan, China	MK001435

Sequences were aligned using MUSCLE (Edgar 2004) integrated in MEGA 11 (Tamura et al. 2021). The best substitution model GTR + Γ was selected using jModelTest 2.1.10 (Darriba et al. 2012). Bayesian Inference (BI) was performed in MrBayes 3.2.7 (Ronquist et al. 2012), based on the selected substitution model. Two runs were performed simultaneously with four Markov chains. The chains were run for 10,000,000 generations and sampled every 1,000 generations. The first 25% of the sampled trees was discarded as burn-in and then the remaining trees were used to estimate Bayesian posterior probabilities (BPP); nodes with BPP values of 0.95 and higher being considered well-supported (Huelsenbeck et al. 2001; Wilcox et al. 2002). Maximum Likelihood (ML) analysis was performed in IQ-TREE 1.6.12 (Nguyen et al. 2015) using the selected substitution model. One thousand bootstrap pseudoreplicates via the ultrafast bootstrap (UFB) approximation algorithm were used to construct a final consensus tree, nodes with UFB values of 95 and above being considered significantly supported (Minh et al. 2013). Uncorrected genetic pairwise distances (p-distances) between species were calculated in MEGA 11 (Tamura et al. 2021) with the pairwise deletion option for handling alignment gaps and missing data.

## ﻿Results

**Figure 2.**
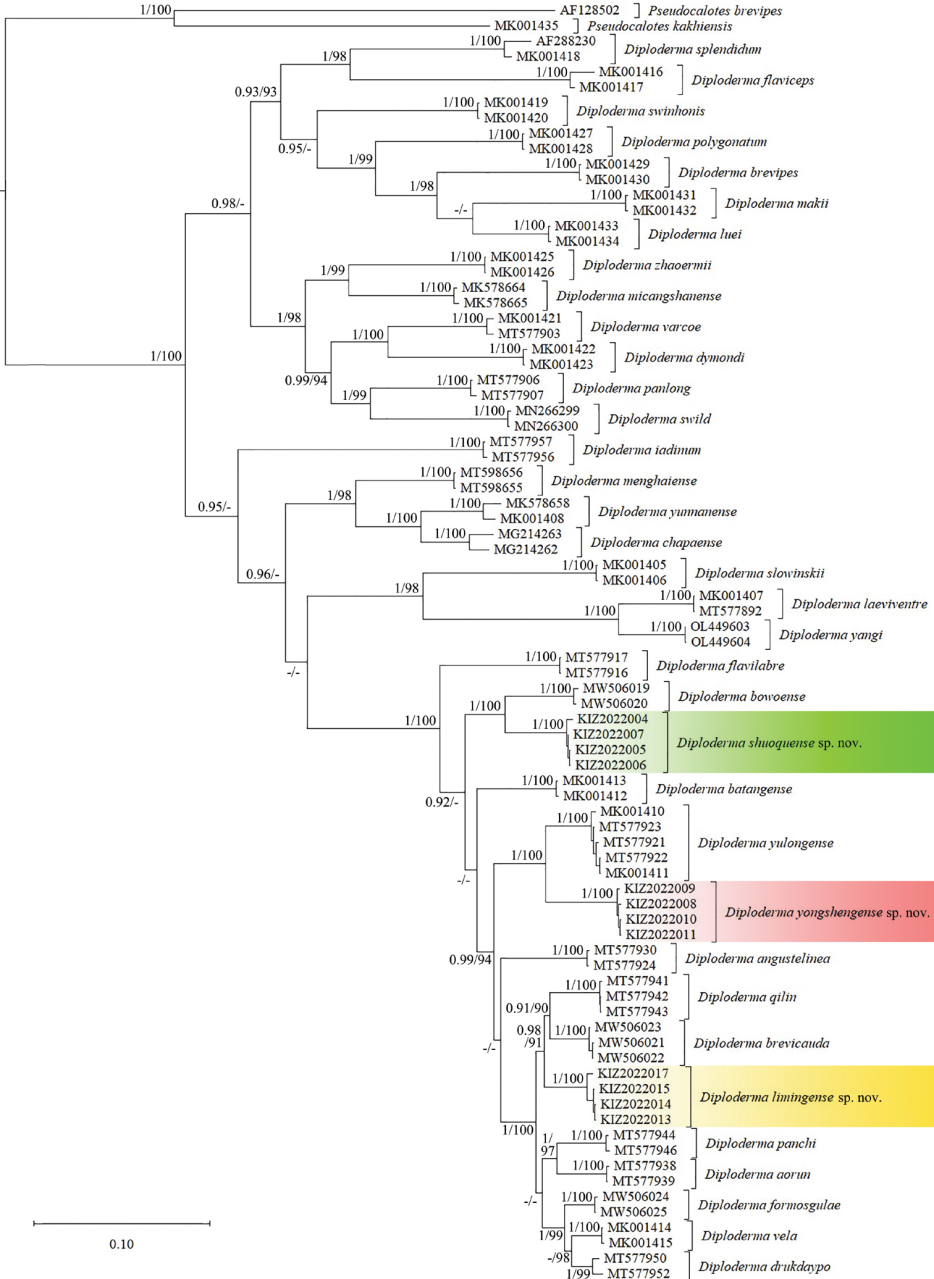
Bayesian phylogram of the genus *Diploderma* inferred from mitochondrial gene ND2 (1031 bp). Numbers before slashes indicate BPP values and numbers after slashes indicate UFB values. The symbol “–” represents the value below 0.90/90.

The obtained sequence alignment is 1031 bp in length. The resulting topologies from BI and ML analyses are consistent (Fig. 2). The specimens from Yulong County formed a clade sister to the clade consisting of *Diplodermaqilin* Wang, Ren, Che & Siler, 2020 and *D.brevicauda* (Manthey, Denzer, Hou & Wang, 2012) with strong support by BI, the specimens from Xiangcheng County formed a clade sister to *D.bowoense* Wang, Gao, Wu, Siler & Che, 2021 with strong support by both BI and ML and the specimens from Yongsheng County formed a clade sister to *D.yulongense* (Manthey, Denzer, Hou & Wang, 2012) with strong support by both BI and ML. The minimum average genetic distance between the specimens from Yulong County and other species of *Diploderma* is 4.1% (between *D.brevicauda*), the minimum average genetic distance between the specimens from Xiangcheng County and other species of *Diploderma* is 6.3% (between *D.bowoense*) and the minimum average genetic distance between the specimens from Yongsheng County and other species of *Diploderma* is 5.8% (between *D.yulongense*) (Suppl. material 1).

### ﻿Taxonomy

#### ﻿ Diploderma limingense sp. nov.

Taxon classificationAnimaliaSquamataAgamidae

E91FF519-A321-5C75-BB09-D91363B15624

https://zoobank.org/3CE0C841-1864-4B05-9D1F-FEB5E193939F

Figs 3 , 4 , 5

##### Holotype.

KIZ2022014, adult male, collected on 21 April 2022 by Shuo Liu from Liming Village, Liming Township, Yulong County, Lijiang City, Yunnan Province, China (27°2′0″N, 99°40′42″E, 2300 m elevation).

##### Paratypes.

KIZ2022013, KIZ2022015, KIZ2022017, three adult males, collecting information the same as the holotype.

##### Etymology.

The specific epithet refers to Liming Township, where the new species was discovered.

##### Diagnosis.

*Diplodermalimingense* sp. nov. can be diagnosed from congeners by a combination of the following morphological characteristics: (1) body size medium, SVL 55.6–56.8 mm in males; (2) tail relatively long, TAL/SVL 1.92–2.09 in males; (3) head moderately wide, HW/HL 0.71–0.74 in males; (4) limbs relatively long, FLL/SVL 0.47–0.52 in males, HLL/SVL 0.74–0.82 in males; (5) MD 45–48; (6) F4S 15–16, T4S 21–22; (7) tympanum concealed; (8) nuchal and dorsal crest scales feebly developed, no skin folds under nuchal and dorsal crest scales in males; (9) distinct transverse gular fold present; (10) ventral head and body scales strongly keeled; (11) ventral head scales heterogeneous in size; (12) gular spot present in males, yellowish-white in life; (13) dorsolateral stripes jagged in males, light yellow in life; (14) ventral surfaces of body, limbs and tail light brick red in males in life; (15) five radial stripes around the eye on each side; (16) inner lips bright yellow, tongue light orange, remaining oral cavity mostly light flesh colour in life.

##### Description of holotype.

Adult male, SVL 56.2 mm; tail relatively long, TAL 117.5 mm, TAL/SVL 2.09; limbs relatively long, FLL 26.5 mm on left side, FLL/SVL 0.47, HLL 41.8 mm on left side, HLL/SVL 0.74. Head relatively robust, HW/HL 0.74, HD/HW 0.85; snout moderately long, SEL/HL 0.36. Rostral elongated, bordered by five small postrostral scales; dorsal head scales heterogeneous, all strongly keeled; indistinct Y-shaped ridge on dorsal snout. Nasal oval, separated from first supralabial by single row of scales; loreals small, keeled; suborbital scale rows 4/3, keeled; canthus rostralis elongated, greatly overlapping with each other; enlarged, keeled scales forming single lateral ridge from posteroinferior eye to posterosuperior tympanum on each side; tympanum concealed under scales; SL 8/8, feebly keeled. Mental pentagonal; IL 9/9; enlarged chin shields 4/5, smooth, first one contacting IL on each side, remaining ones separated from IL by two rows of small scales; ventral head scales homogeneous in size, smooth or weakly keeled; distinct transverse gular fold present; gular pouch weakly developed.

Distinct shoulder fold present; dorsal body scales heterogeneous in size and shape, all keeled, tip pointing backwards; axillary scales much smaller than remaining dorsals; enlarged dorsal scales roughly forming four longitudinal rows from neck to pelvis on each side of body. Nuchal and dorsal crests continuous, scales of nuchal and dorsal crests approximately same in size and shape; no skin fold under nuchal and dorsal crests; MD 45. Dorsal limb scales strongly keeled, homogeneous on fore-limbs and heterogeneous on hind limbs; F4S 15/16, T4S 22/22. Ventral body scales approximately parallel, almost homogeneous, all strongly keeled, VN 63. Ventral limb scales parallel, small on fore-limbs and larger on hind limbs, all strongly keeled. Tail scales all strongly keeled, ventral tail scales larger than dorsal tail scales.

##### Colouration of holotype in life.

Dorsal surface of head brownish-grey. A distinct black transverse band anteriorly and an indistinct black transverse band posteriorly present between orbits on dorsal surface of head. Lateral surfaces of head brownish-grey. Five brownish-black radial stripes around eye on each side. Upper lips greyish-white. Inner lips bright yellow, tongue light orange, remaining oral cavity mostly light flesh colour.

Dorsal surface of body brown. A light yellow jagged dorsolateral stripe present from neck to pelvis on each side of body. Some brownish-black triangular patches distributed along vertebral line between dorsolateral stripes from neck to base of tail, all of which pointing posteriorly. Some yellowish-white spots scattered below dorsolateral stripe on each side of body. Dorsal surfaces of limbs greyish-brown with indistinct dark transverse bands. Dorsal surface of tail brownish-grey with some indistinct dark transverse bands.

Ventral surface of head greyish-white. A roughly triangular, yellowish-white gular spot present on posterior central part, many grey stripes forming reticulated pattern present on other region of ventral head. Ventral surfaces of body, limbs and tail light brick red with no patterns.

##### Variations.

The variations of morphological character of the type series are provided in Table 2. The variations of colouration in life are very small: the paratype KIZ2022013 has few yellowish-white spots below dorsolateral stripe on each side of body, except for this, all other paratypes closely resemble the holotype.

**Table 2.** Morphological data of the type series of *Diplodermalimingense* sp. nov. Morphometric measurements are in mm. For measurement methods and abbreviations, see the Materials and methods section.

	KIZ2022014 Holotype ♂	KIZ2022013 Paratype ♂	KIZ2022015 Paratype ♂	KIZ2022017 Paratype ♂
SVL	56.2	56.8	55.6	56.1
TAL	117.5	110.1	113.5	107.7
HL	18.0	18.5	18.0	18.0
HW	13.3	13.2	13.2	13.1
HD	11.3	11.2	11.0	11.4
SEL	6.5	6.7	6.3	6.9
FLL	26.5	27.5	28.7	27.6
HLL	41.8	44.9	45.6	43.4
T4L	10.7	10.9	11.2	9.9
TRL	25.1	24.2	23.6	24.9
TAL/SVL	2.09	1.94	2.04	1.92
SEL/HL	0.36	0.36	0.35	0.38
HW/HL	0.74	0.71	0.73	0.73
HD/HW	0.85	0.85	0.83	0.87
FLL/SVL	0.47	0.48	0.52	0.49
HLL/SVL	0.74	0.79	0.82	0.77
TRL/SVL	0.45	0.43	0.42	0.44
SL	8/8	8/8	8/9	9/9
IL	9/9	8/9	10/8	9/9
NSL	2/1	1/1	1/1	1/1
MD	45	45	47	48
F4S	15/16	16/16	15/16	16/16
T4S	22/22	22/22	22/21	21/21
SOR	4/3	3/4	3/3	3/3
VN	63	58	59	63

##### Comparisons.

From species of *Diploderma* which are only distributed on East Asian islands, *Diplodermalimingense* sp. nov. differs from *D.brevipes* (Gressitt, 1936), *D.luei* (Ota, Chen & Shang, 1998), *D.makii* (Ota, 1989), *D.polygonatum* Hallowell, 1861 and *D.swinhonis* (Günther, 1864) by the presence of a transverse gular fold (vs. absence).

From species of *Diploderma* which are distributed on mainland, but relatively distant from that of *Diplodermalimingense* sp. nov., *Diplodermalimingense* sp. nov. differs from *D.chapaense* (Bourret, 1937), *D.fasciatum* (Mertens, 1926), *D.hamptoni* (Smith, 1935), *D.menghaiense* Liu, Hou, Wang, Ananjeva & Rao, 2020, *D.micangshanense* (Song, 1987), *D.ngoclinense* (Ananjeva, Orlov & Nguyen, 2017) and *D.yunnanense* (Anderson, 1878) by the presence of a transverse gular fold (vs. absence); from *D.dymondi* (Boulenger, 1906), *D.varcoae* (Boulenger, 1918), by having concealed tympana (vs. exposed); from *D.grahami* (Stejneger, 1924) by having a much longer tail (TAL/SVL 1.92–2.09 vs. 1.64) and a distinct transverse gular fold (vs. feeble); and from *D.splendidum* (Barbour & Dunn, 1919) by having jagged dorsolateral stripes in males (vs. smooth).

From species of *Diploderma* which occupy distributions relatively close to that of *Diplodermalimingense* sp. nov. in the Hengduan Mountain Region, *Diplodermalimingense* sp. nov. differs from *D.panlong* Wang, Che & Siler, 2020, *D.slowinskii*, (Rao, Vindum, Ma, Fu & Wilkinson, 2017) and *D.swild* Wang, Wu, Jiang, Chen, Miao, Siler & Che, 2019 by having concealed tympana (vs. exposed); from *D.angustelinea* Wang, Ren, Wu, Che & Siler, 2020, *D.aorun* Wang, Jiang, Zheng, Xie, Che & Siler, 2020, *D.bowoense*, *D.batangense* (Li, Deng, Wu & Wang, 2001), *D.flavilabre* Wang, Che & Siler, 2020, *D.formosgulae* Wang, Gao, Wu, Dong, Shi, Qi, Siler & Che, 2021, *D.iadinum* (Wang, Jiang, Siler & Che, 2016), *D.laeviventre* (Wang, Jiang, Siler & Che, 2016), *D.yangi* Wang, Zhang & Li, 2022, *D.yulongense* and *D.zhaoermii* (Gao & Hou, 2002) by having a yellowish-white gular spot in males in life (vs. chartreuse, blue, green, lilac, orange or yellow); from *D.drukdaypo* (Wang, Ren, Jiang, Zou, Wu, Che & Siler, 2019) by having strongly keeled ventral scales of body (vs. smooth or weakly keeled); from *D.flaviceps* (Barbour & Dunn, 1919) by the presence of a colourful gular spot in males in life (vs. absence) and no skin fold under dorsal and nuchal crests in males (vs. strongly developed and erected); from *D.panchi* Wang, Zheng, Xie, Che & Siler, 2020 by having bright yellow inner lips in life (vs. inner lips flesh colour); and from *D.vela* (Wang, Jiang & Che, 2015) by having feebly developed crests without strongly erected crest scales or skin fold in males in life (vs. distinctively erected crest scales on continuous, well-developed skin fold).

**Figure 3.**
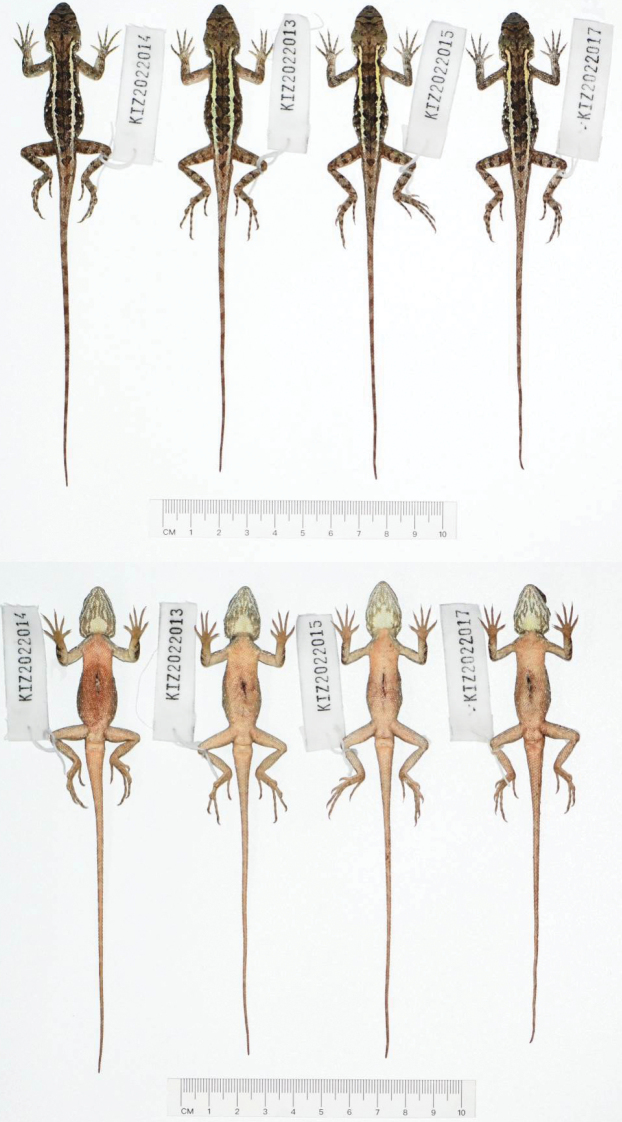
Dorsal view (top) and ventral view (bottom) of type series of *Diplodermalimingense* sp. nov. in preservative.

**Figure 4.**
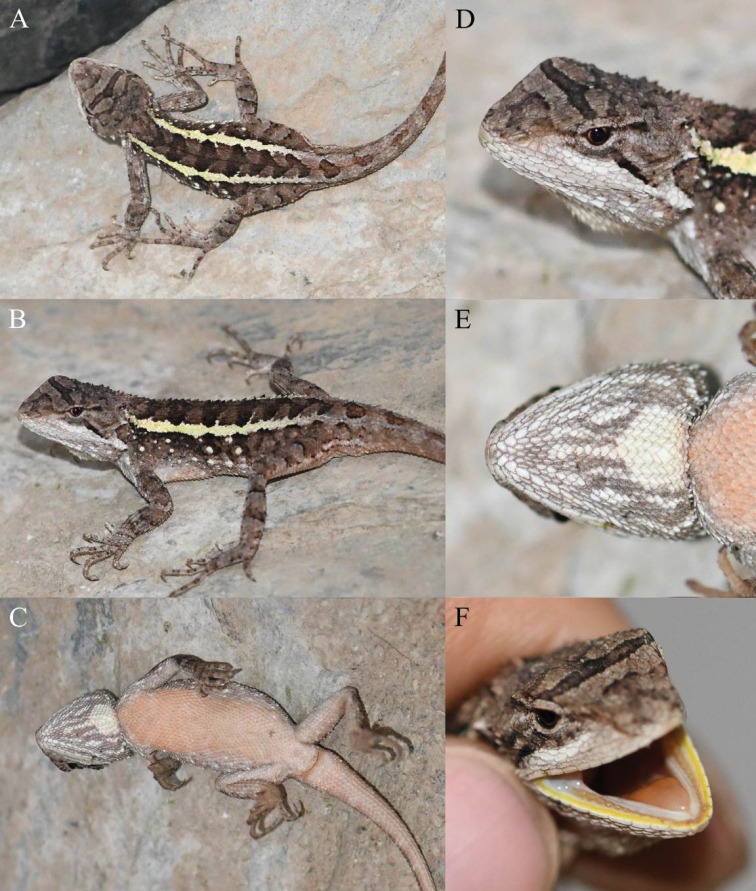
Holotype (KIZ2022014) of *Diplodermalimingense* sp. nov. in life **A** dorsal view **B** lateral view **C** ventral view **D** close-up view of the dorsolateral side of the head **E** close-up view of the ventral side of the head **F** close-up view of the oral cavity.

**Figure 5.**
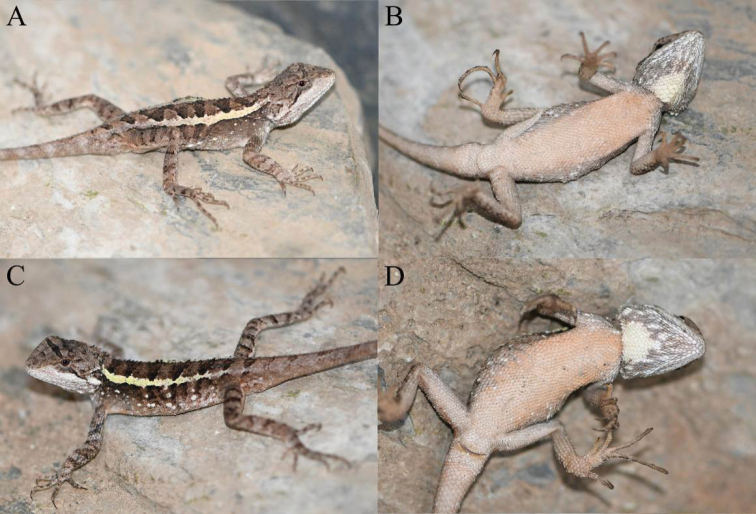
Paratypes of *Diplodermalimingense* sp. nov. in life **A** dorsolateral view of the paratype KIZ2022013 **B** ventral view of the paratype KIZ2022013 **C** dorsolateral view of the paratype KIZ2022015 **D** ventral view of the paratype KIZ2022015.

*Diplodermalimingense* sp. nov. is phylogenetically sister to *D.qilin* and *D.brevicauda*, but *Diplodermalimingense* sp. nov. can be differentiated from *D.qilin* by having bright yellow inner lips and light orange tongue in life (vs. both inner lips and tongue light flesh colour) and from *D.brevicauda* by having a relatively longer tail in males (TAL/SVL 1.92–2.09 vs. 1.40–1.84) and more mid-dorsal crest scales (MD 45–48 vs. 34–43).

##### Distribution.

This species is known only from the type locality, Liming Township, Yulong County, Lijiang City, Yunnan Province, China (Fig. 1).

##### Natural history.

All specimens were collected between 9 and 11 a.m. on the ground in coniferous and broad-leaved mixed forest and there was no water body nearby (Fig. 12A, B). No female or juvenile was found. The population density of this species was moderate and as the habitats of this species not being threatened. According to IUCN Criteria, we recommend listing this new species as Least Concern (LC).

#### ﻿ Diploderma shuoquense sp. nov.

Taxon classificationAnimaliaSquamataAgamidae

3F48C822-0334-5F99-B5A6-82F489182255

https://zoobank.org/53A4844E-ADBF-4BE0-A924-355D1534019E

Figs 6 , 7 , 8

##### Holotype.

KIZ2022004, adult male, collected on 23 April 2022 by Shuo Liu from the Shuoqu River Valley, Qingde Town, Xiangcheng County, Ganzi Prefecture, Sichuan Province, China (28°48′50″N, 99°49′47″E, 2700 m elevation).

##### Paratypes.

KIZ2022005–KIZ2022007, three adult males, collecting information the same as the holotype.

##### Etymology.

The specific epithet refers to the Shuoqu River, by which the new species was discovered.

##### Diagnosis.

*Diplodermashuoquense* sp. nov. can be diagnosed from congeners by a combination of the following morphological characteristics: (1) body size small, SVL 48.2–52.3 mm in males; (2) tail moderately long, TAL/SVL 1.87–1.97 in males; (3) limbs moderately long, FLL/SVL 0.45–0.49 in males, HLL/SVL 0.69–0.74 in males; (4) head moderately wide, HW/HL 0.72–0.74 in males; (5) MD 34–40; (6) F4S 13–16, T4S 19–21; (7) tympanum concealed; (8) nuchal and dorsal crest scales feebly developed, not distinctively erected or raised on skin folds in males; (9) distinct transverse gular fold present; (10) ventral head scales smooth or weakly keeled and ventral body scales strongly keeled; (11) ventral head scales homogeneous in size; (12) no distinct gular spot in males; (13) dorsolateral stripes jagged in males, yellowish-white or greyish-white in life; (14) 8–10 radial stripes around the eye on each side; (15) oral cavity, inner lips and tongue pink in life.

##### Description of holotype.

Adult male, SVL 52.3 mm; tail moderately long, TAL 98.3 mm, TAL/SVL 1.88; limbs moderately long, FLL 23.4 mm on left side, FLL/SVL 0.45, HLL 36.6 mm on left side, HLL/SVL 0.70. Head relatively robust, HW/HL 0.74, HD/HW 0.82; snout relatively short, SEL/HL 0.34. Rostral rectangular, bordered by six small postrostral scales; dorsal head scales heterogeneous, all strongly keeled; indistinct Y-shaped ridge on dorsal snout. Nasal oval, separated from first supralabial by single row of scales; loreals small, keeled; suborbital scale rows 3/4, keeled; canthus rostralis elongated, greatly overlapping with each other; enlarged, keeled scales forming single lateral ridge from posteroinferior eye to posterosuperior tympanum on each side; tympanum concealed under scales; SL 10/10, feebly keeled. Mental pentagonal; IL 9/9; enlarged chin shields 6/5, smooth, first one contacting IL on left side and first two contacting IL on right side, remaining ones separated from IL by one or two rows of small scales; ventral head scales homogeneous in size, smooth or weakly keeled; distinct transverse gular fold present; gular pouch weakly developed.

Distinct shoulder fold present; dorsal body scales heterogeneous in size and shape, all keeled, tip pointing backwards; axillary scales much smaller than remaining dorsals; enlarged dorsal scales roughly forming four or five longitudinal rows from neck to pelvis on each side of body. Nuchal and dorsal crests feebly developed, slightly raised compared to dorsals, not erect; no skin fold under nuchal and dorsal crests; MD 40. Dorsal limb scales strongly keeled, homogeneous; F4S 15/16, T4S 21/20. Ventral body scales approximately parallel, almost homogeneous, all strongly keeled, VN 61. Ventral limb scales parallel, almost homogeneous, approximately equal in size to ventrals, all strongly keeled. Tail scales all strongly keeled, ventral tail scales slightly larger than dorsal tail scales.

##### Colouration of holotype in life.

Dorsal surface of head grey. Two distinct black transverse bands present between orbits on dorsal surface of head and two indistinct greyish-black transverse bands present on dorsal surface of snout. Lateral surfaces of head greyish-white. Ten black radial stripes around eye on each side. Upper lips light orange. Oral cavity, inner lips and tongue pink.

Dorsal surface of body greyish-black. A light yellowish-white dorsolateral longitudinal stripe with strongly jagged upper edge and relatively straight lower edge present on each side of body from occipital region to pelvis. Some indistinct dark and light transverse bands present between two dorsolateral stripes. Some white spots scattered below dorsolateral stripe on each side of body. Dorsal surfaces of limbs dark grey. Some irregular, greyish-white transverse bands present on dorsal surfaces of limbs. Dorsal surface of tail grey with some very indistinct dark transverse bands.

Ventral surface of head white with distinct black vermiculate stripes. A little yellowish colouration present on centre of gular pouch. Ventral surfaces of body, limbs and tail white with no patterns.

##### Variations.

The variations of morphological character of the type series are provided in Table 3. The variations of colouration in life are as follows: the paratypes resemble the holotype in most aspects, except that the dorsal colouration is darker in the paratype KIZ2022007, the light orange colouration on upper lips is more indistinct in the paratypes KIZ2022005 and KIZ2022006, there is no yellowish colouration on the centre of the gular pouch in the paratypes KIZ2022006 and KIZ2022007 and there is some yellowish colouration on the chest in the paratype KIZ2022005.

**Table 3.** Morphological data of the type series of *Diplodermashuoquense* sp. nov. Morphometric measurements are in mm. For measurement methods and abbreviations, see the Materials and methods section.

	KIZ2022004 Holotype ♂	KIZ2022005 Paratype ♂	KIZ2022006 Paratype ♂	KIZ2022007 Paratype ♂
SVL	52.3	48.2	48.4	50.5
TAL	98.3	90.3	95.4	96.3
HL	16.7	14.3	15.4	15.7
HW	12.4	10.5	11.1	11.3
HD	10.2	8.7	9.2	9.8
SEL	5.7	5.4	5.4	5.6
FLL	23.4	22.6	23.5	23.7
HLL	36.6	33.4	36.0	36.4
T4L	8.6	7.8	8.7	8.6
TRL	23.6	22.1	22.5	21.3
TAL/SVL	1.88	1.87	1.97	1.91
SEL/HL	0.34	0.38	0.35	0.36
HW/HL	0.74	0.73	0.72	0.72
HD/HW	0.82	0.83	0.83	0.87
FLL/SVL	0.45	0.47	0.49	0.47
HLL/SVL	0.70	0.69	0.74	0.72
TRL/SVL	0.45	0.46	0.46	0.42
SL	10/10	10/10	9/9	9/8
IL	9/9	11/11	10/10	10/11
NSL	1/1	1/1	1/1	1/1
MD	40	34	39	34
F4S	15/16	14/13	15/15	15/14
T4S	21/20	20/19	20/20	20/19
SOR	3/4	4/4	3/4	4/4
VN	61	59	57	56

##### Comparisons.

From species of *Diploderma* which are only distributed on East Asian Islands, *Diplodermashuoquense* sp. nov. differs from *D.brevipes*, *D.luei*, *D.makii*, *D.polygonatum* and *D.swinhonis* by the presence of a transverse gular fold (vs. absence).

From species of *Diploderma* which are distributed on mainland, but relatively distant from that of *Diplodermashuoquense* sp. nov., *Diplodermashuoquense* sp. nov. differs from *D.chapaense*, *D.fasciatum*, *D.hamptoni*, *D.menghaiense*, *D.micangshanense*, *D.ngoclinense* and *D.yunnanense* by the presence of a transverse gular fold (vs. absence); from *D.dymondi*, *D.varcoae*, by having concealed tympana (vs. exposed); from *D.grahami* by having a much longer tail (TAL/SVL 1.87–1.97 vs. 1.64) and a distinct transverse gular fold (vs. feeble); and from *D.splendidum* by having jagged dorsolateral stripes in males (vs. smooth).

From species of *Diploderma* which occupy distributions relatively close to that of *Diplodermashuoquense* sp. nov. in the Hengduan Mountain Region, *Diplodermashuoquense* sp. nov. differs from *D.panlong*, *D.slowinskii* and *D.swild* by having concealed tympana (vs. exposed); from *D.angustelinea*, *D.aorun*, *D.batangense*, *D.flavilabre*, *D.formosgulae*, *D.iadinum*, *D.laeviventre*, *D.yangi*, *D.yulongense* and *D.zhaoermii* by the absence of a distinct gular spot in males in life (vs. presence of a distinct colourful gular spot); from *D.brevicauda* by having a relatively longer tail in males (TAL/SVL 1.87–1.97 vs. 1.40–1.84) and pink inner lips and tongue in life (vs. inner lips light yellow and tongue light orange); from *D.drukdaypo* by having strongly keeled ventral scales of body (vs. smooth or weakly keeled); from *D.flaviceps* by the presence of distinct radial stripes around the eyes (vs. absence) and the absence of a skin fold under dorsal crest in males in life (vs. presence); from *D.panchi* by having less mid-dorsal crest scales (MD 34–40 vs. 42–46) and smooth or weakly keeled ventral scales of head (vs. distinctively keeled); from *D.qilin* by having a relatively shorter tail in males (TAL/SVL 1.87–1.97 vs. 2.01–2.18); and from *D.vela* by having feebly developed crests without strongly erected crest scales or skin fold in males in life (vs. distinctively erected crest scales on continuous, well-developed skin fold).

**Figure 6.**
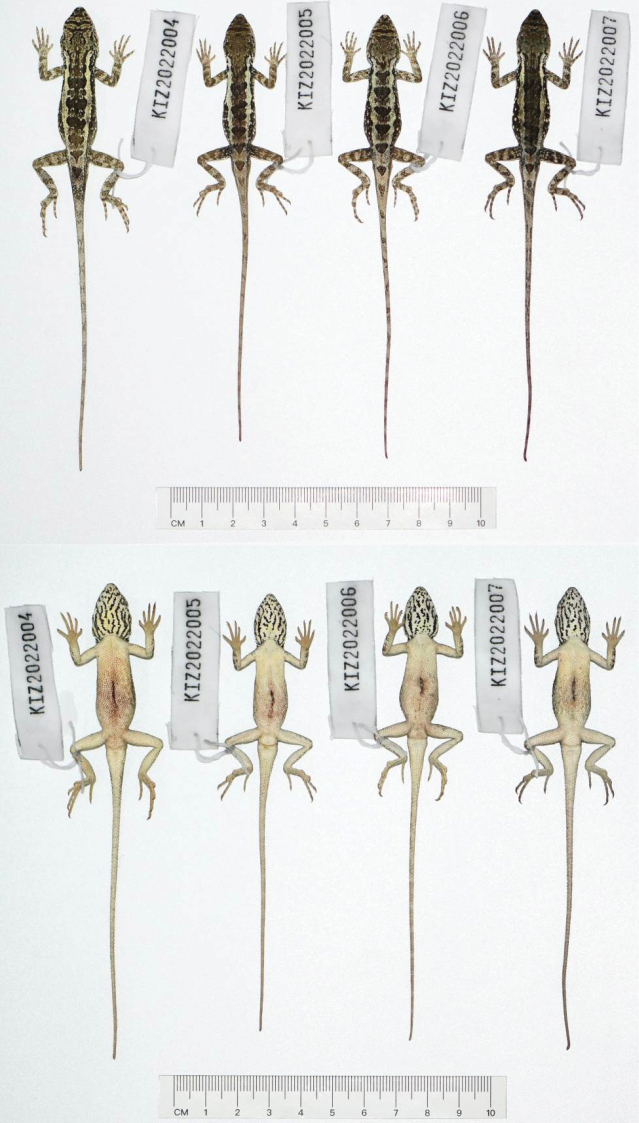
Dorsal view (top) and ventral view (bottom) of type series of *Diplodermashuoquense* sp. nov. in preservative.

**Figure 7.**
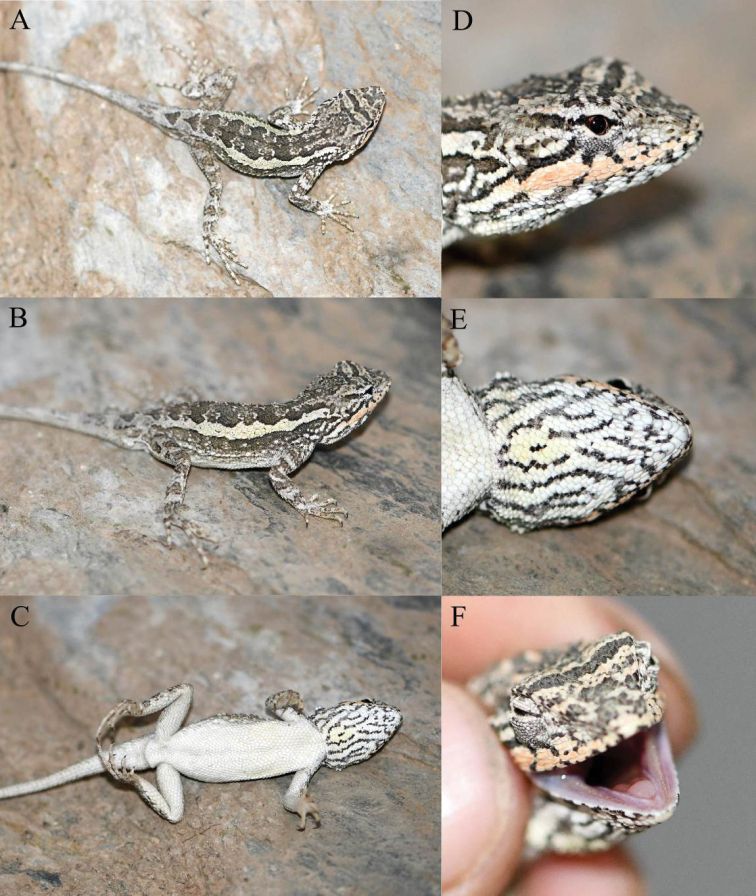
Holotype (KIZ2022004) of *Diplodermashuoquense* sp. nov. in life **A** dorsal view **B** lateral view **C** ventral view **D** close-up view of the lateral side of the head **E** close-up view of the ventral side of the head **F** close-up view of the oral cavity.

**Figure 8.**
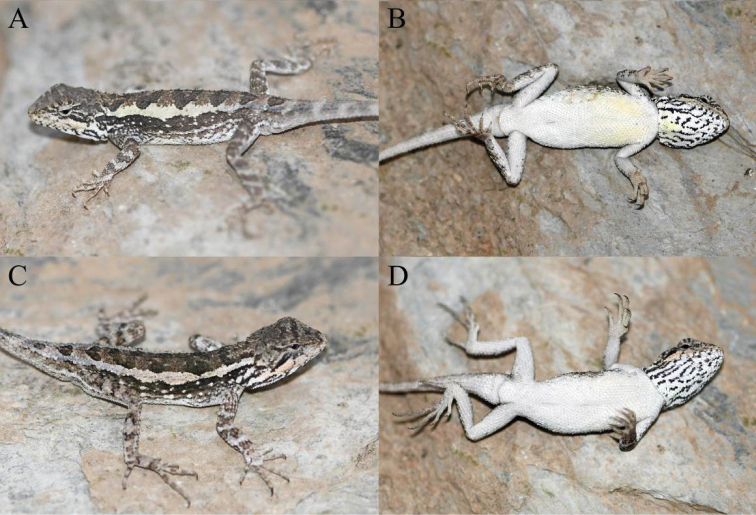
Paratypes of *Diplodermashuoquense* sp. nov. in life **A** dorsolateral view of the paratype KIZ2022005 **B** ventral view of the paratype KIZ2022005 **C** dorsolateral view of the paratype KIZ2022007 **D** ventral view of the paratype KIZ2022007.

*Diplodermashuoquense* sp. nov. is phylogenetically sister to *D.bowoense*, but *Diplodermashuoquense* sp. nov. can be differentiated from the latter by the absence of a light chrome orange gular spot in males in life (vs. presence) and having a wider head (HW/HL 0.72–0.74 vs. 0.65–0.71) and smooth or weakly keeled ventral scales of head (vs. distinctively keeled).

*Diplodermashuoquense* sp. nov. differs from *Diplodermalimingense* sp. nov. by having a smaller body size in males (SVL 48.2–52.3 mm vs. 55.6–56.8 mm), vermiculate stripes covering the whole ventral head (vs. stripes not reaching the centre of gular pouch), white ventral surfaces of body, limbs and tail in males in life (vs. light brick red), pink inner lips and tongue in life (vs. inner lips bright yellow, tongue light orange) and more radial stripes around the eyes (8–10 vs. five on each side).

##### Distribution.

This species is known only from the type locality, Qingde Town, Xiangcheng County, Ganzi Prefecture, Sichuan Province, China (Fig. 1).

##### Natural history.

This species is terrestrial, inhabiting the hot-dry valley. There are many thorny shrubs and some rock piles at the type locality (Fig. 12C, D). All specimens were collected between 1 and 3 p.m. when they were basking on rock piles, no female or juvenile being found. We found many locusts at the type locality, which may be the main prey of this species; however, the population density of this species was very low and the habitats at the type locality being threatened by human activities. According to IUCN Criteria, we recommend listing this new species as Vulnerable (VU).

#### ﻿ Diploderma yongshengense sp. nov.

Taxon classificationAnimaliaSquamataAgamidae

4056C64F-E942-53E8-9913-8EFFB14942E1

https://zoobank.org/855A40FC-484D-430F-A50E-077512BA9BE8

Figs 9 , 10 , 11

##### Holotype.

KIZ2022009, adult male, collected on 24 April 2022 by Shuo Liu from the Jinsha River Valley, Songping Township, Yongsheng County, Lijiang City, Yunnan Province, China (27°2′2″N, 100°28′16″E, 1700 m elevation).

##### Paratypes.

KIZ2022008, KIZ2022010–KIZ2022011, three adult males, collecting information the same as the holotype.

##### Etymology.

The specific epithet refers to Yongsheng County, where the new species was discovered.

##### Diagnosis.

*Diplodermayongshengense* sp. nov. can be diagnosed from congeners by a combination of the following morphological characteristics: (1) body size moderate, SVL 56.5–58.5 mm in males; (2) tail long, TAL/SVL 2.02–2.20 in males; (3) limbs relatively long, FLL/SVL 0.48–0.51 in males, HLL/SVL 0.79–0.87 in males; (4) head moderately wide, HW/HL 0.66–0.75 in males; (5) MD 38–41; (6) F4S 16–19, T4S 22–25; (7) tympanum concealed; (8) nuchal and dorsal crests moderately developed on weak skin folds in males; (9) distinct transverse gular fold present; (10) ventral scales of head and body strongly keeled; (11) ventral head scales heterogeneous in size; (12) gular spot present in males, blue or green in life; (13) dorsolateral stripes jagged in males, light yellow in life; (14) radial stripes around the eyes indistinct; (15) oral cavity, inner lips and tongue light flesh colour in life.

##### Description of holotype.

Adult male, SVL 58.5 mm; tail long, TAL 128.7 mm, TAL/SVL 2.20; limbs relatively long, FLL 27.9 mm on left side, FLL/SVL 0.48, HLL 46.5 mm on left, HLL/SVL 0.79. Head relatively robust, HW/HL 0.75, HD/HW 0.87; snout moderately long, SEL/HL 0.37. Rostral elongated, bordered by five small postrostral scales; dorsal head scales heterogeneous, all strongly keeled; distinct Y-shaped ridge on dorsal snout. Nasal oval, separated from first supralabial by single row of scales; loreals small, keeled; suborbital scale rows 4/4, keeled; canthus rostralis elongated, greatly overlapping with each other; enlarged, keeled scales forming single lateral ridge from posteroinferior eye to posterosuperior tympanum on each side; tympanum concealed under scales; SL 9/9, feebly keeled. Mental pentagonal; IL 11/10; enlarged chin shields 5/5, smooth, first one contacting IL on each side, remaining ones separated from IL by two rows of small scales; ventral head scales heterogeneous in size with the ones on the centre of gular pouch largest, all strongly keeled; distinct transverse gular fold present; gular pouch well developed.

Distinct shoulder fold present; dorsal body scales heterogeneous in size and shape, all keeled, tip pointing backwards; axillary scales much smaller than remaining dorsals; enlarged dorsal scales irregularly scattered on lateral surface of body. Nuchal crest scales approximately same in size and shape as dorsal crest scales; moderately developed skin fold under nuchal crest and feeble skin fold under dorsal crest; MD 38. Dorsal limb scales strongly keeled, mostly homogeneous, except a few enlarged, conical scales on postaxial thighs; F4S 17/16, T4S 23/23. Ventral body scales approximately parallel, almost homogeneous, all strongly keeled, VN 59. Ventral limb scales parallel, almost homogeneous, approximately equal in size to ventrals, all strongly keeled. Tail scales all strongly keeled, ventral tail scales larger than dorsal tail scales.

##### Colouration of holotype in life.

Dorsal surface of head brown with no transverse bands. Lateral surfaces of head brownish-white. No radial stripes present around eyes, only two brownish-black stripes present behind eye on each side. Oral cavity, inner lips and tongue light flesh colour.

Dorsal surface of body brown. A light yellow dorsolateral longitudinal stripe with relatively straight upper edge and strongly jagged lower edge present on each side of body from occipital region to pelvis. Some brownish-black transverse bands present between two dorsolateral stripes. Some light yellow spots scattered below dorsolateral stripe on each side of body. Dorsal surfaces of limbs greyish-brown. Some indistinct dark transverse bands present on dorsal surfaces of limbs. Dorsal surface of tail brownish-grey with some indistinct dark transverse bands.

Ventral surface of head yellowish-white. A triangular, light yellow edged light blue gular spot present on posterior central part, indistinct brown stripes present on other region of ventral head. Ventral surfaces of body, limbs and tail white with no patterns.

##### Variations.

The variations of morphological character of the type series are provided in Table 4. The variations of colouration in life are as follows: the paratypes resemble the holotype in most aspects, except that there are indistinct transverse bands on the dorsal surface of the head in all paratypes; the gular spot is light green in the paratypes KIZ2022008 and KIZ2022010; the dorsal colouration is darker, the stripes on the ventral surface of head are more distinct in the paratypes KIZ2022008 and KIZ2022011; and there are some brown speckles on the ventral surfaces of body, limbs and tail in the paratype KIZ2022008.

**Table 4.** Morphological data of the type series of *Diplodermayongshengense* sp. nov. Morphometric measurements are in mm. For measurement methods and abbreviations, see the Materials and methods section.

	KIZ2022008 Paratype ♂	KIZ2022009 Holotype ♂	KIZ2022010 Paratype ♂	KIZ2022011 Paratype ♂
SVL	56.5	58.5	56.7	57.6
TAL	117.2	128.7	114.5	123.0
HL	17.9	18.7	17.0	18.8
HW	12.8	14.1	12.1	12.5
HD	11.1	11.0	10.6	11.3
SEL	6.6	6.9	6.3	6.9
FLL	28.6	27.9	27.8	27.4
HLL	49.1	46.5	45.5	47.9
T4L	12.6	11.8	11.3	13.1
TRL	24.3	27.0	24.9	26.1
TAL/SVL	2.07	2.20	2.02	2.14
SEL/HL	0.37	0.37	0.37	0.37
HW/HL	0.72	0.75	0.71	0.66
HD/HW	0.87	0.78	0.88	0.90
FLL/SVL	0.51	0.48	0.49	0.48
HLL/SVL	0.87	0.79	0.80	0.83
TRL/SVL	0.43	0.46	0.44	0.45
SL	10/10	9/9	8/8	9/9
IL	11/10	11/10	10/12	10/10
NSL	1/1	1/1	1/1	1/1
MD	41	38	41	39
F4S	17/18	17/16	19/18	16/17
T4S	22/23	23/23	25/24	24/24
SOR	4/4	4/4	4/4	4/4
VN	55	59	58	54

##### Comparisons.

From species of *Diploderma* which are only distributed on East Asian Islands, *Diplodermayongshengense* sp. nov. differs from *D.brevipes*, *D.luei*, *D.makii*, *D.polygonatum* and *D.swinhonis* by the presence of a transverse gular fold (vs. absence).

From species of *Diploderma* which are distributed on mainland, but relatively distant from that of *Diplodermayongshengense* sp. nov., *Diplodermayongshengense* sp. nov. differs from *D.chapaense*, *D.fasciatum*, *D.hamptoni*, *D.menghaiense*, *D.micangshanense*, *D.ngoclinense* and *D.yunnanense* by the presence of a transverse gular fold (vs. absence); from *D.dymondi*, *D.varcoae*, by having concealed tympana (vs. exposed); from *D.grahami* by having a much longer tail (TAL/SVL 2.02–2.20 vs. 1.64) and a distinct transverse gular fold (vs. feeble); and from *D.splendidum* by having jagged dorsolateral stripes in males (vs. smooth).

From species of *Diploderma* which occupy distributions relatively close to that of *Diplodermayongshengense* sp. nov. in the Hengduan Mountain Region, *Diplodermayongshengense* sp. nov. differs from *D.panlong*, *D.slowinskii* and *D.swild* by having concealed tympana (vs. exposed); from *D.drukdaypo* and *D.vela* by the presence of a colourful gular spot in males in life (vs. absence); from *D.angustelinea*, *D.bowoense*, *D.brevicauda*, *D.formosgulae*, *D.laeviventre*, *D.qilin* and *D.zhaoermii* by having a blue or green gular spot in males in life (vs. chartreuse, lilac, orange or yellow); from *D.aorun* by having less distinct radial stripes around the eyes (vs. more distinct), less distinct stripes on the ventral surface of head (vs. more distinct speckles or vermiculated patterns) and heterogeneous ventral head scales (vs. homogeneous); from *D.batangense* by having white ventral surface of body in males in life (vs. yellow); from *D.flaviceps* by the presence of a colourful gular spot in males in life (vs. absence); from *D.flavilabre* by having light flesh coloured inner lips in life (vs. yellow); from *D.iadinum* by having brown dorsal ground colouration in males in life (vs. emerald green); from *D.panchi* by having less mid-dorsal crest scales (MD 38–41 vs. 42–46) and heterogeneous ventral head scales (vs. homogeneous); and from *D.yangi* by having jagged dorsolateral stripes in males (vs. smooth).

*Diplodermayongshengense* sp. nov. is phylogenetically sister to *D.yulongense*, but *Diplodermayongshengense* sp. nov. can be differentiated from the latter by having a blue or green gular spot in males in life (vs. chartreuse or opaline green), more distinct stripes on the ventral surface of head (vs. less distinct), white ventral and ventrolateral surface of body in males in life (vs. green) and light yellow dorsolateral stripes and enlarged scales on each side of body in males in life (vs. greenish-yellow).

*Diplodermayongshengense* sp. nov. differs from *Diplodermalimingense* sp. nov. by having less mid-dorsal crest scales (MD 38–41 vs. 45–48), a blue or green gular spot in males in life (vs. yellowish-white), white ventral surfaces of body, limbs and tail in males in life (vs. light brick red) and light flesh coloured inner lips and tongue in life (vs. inner lips bright yellow, tongue light orange).

**Figure 9.**
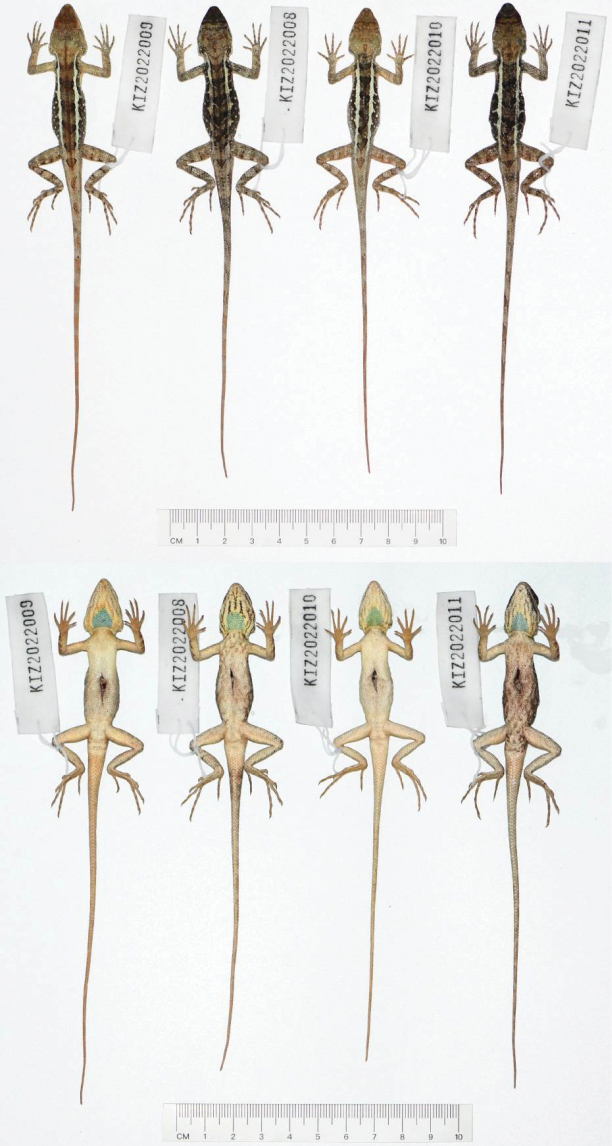
Dorsal view (top) and ventral view (bottom) of type series of *Diplodermayongshengense* sp. nov. in preservative.

**Figure 10.**
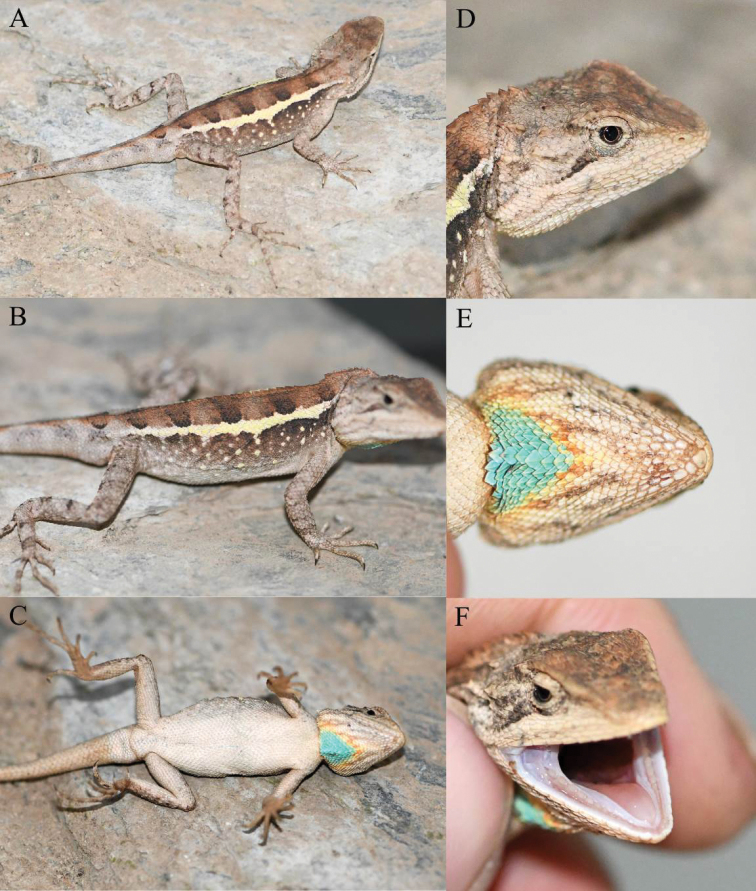
Holotype (KIZ2022009) of *Diplodermayongshengense* sp. nov. in life **A** dorsolateral view **B** lateral view **C** ventral view **D** close up-view of the lateral side of the head **E** close-up view of the ventral side of the head **F** close-up view of the oral cavity.

**Figure 11.**
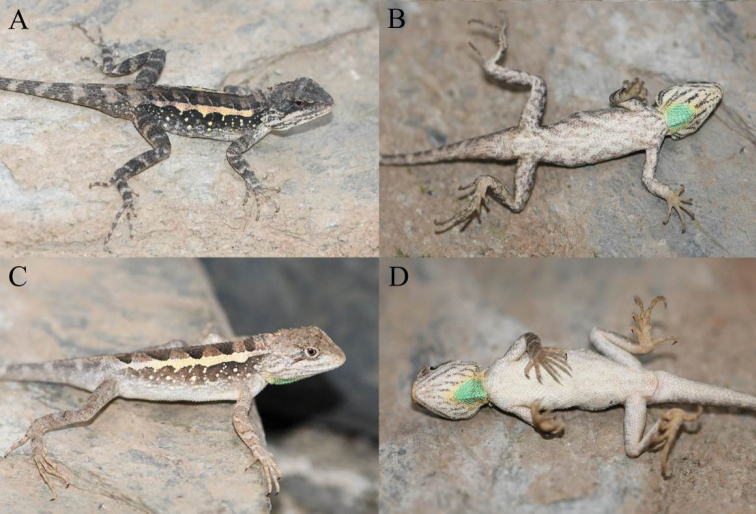
Paratypes of *Diplodermayongshengense* sp. nov. in life **A** dorsolateral view of the paratype KIZ2022008 **B** ventral view of the paratype KIZ2022008 **C** lateral view of the paratype KIZ2022010 **D** ventral view of the paratype KIZ2022010.

*Diplodermayongshengense* sp. nov. differs from *Diplodermashuoquense* sp. nov. by having a larger body size in males (SVL 56.5–58.5 vs. 48.2–52.3), a relatively longer tail in males (TAL/SVL 2.02–2.20 vs. 1.87–1.97), relatively longer hind limbs in males (HLL/SVL 0.79–0.87 vs. 0.69–0.74), more subdigital lamellae of fourth toe (22–25 vs. 19–21) and strongly keeled ventral scales of head (vs. smooth or weakly keeled) and the presence of a distinct colourful gular spot in males in life (vs. absence).

##### Distribution.

This species is presently known from Yongsheng and Ninglang counties, Lijiang City, Yunnan Province, China, it probably occurs in adjacent Muli County, Sichuan Province, China (Fig. 1).

##### Natural history.

**Figure 12.**
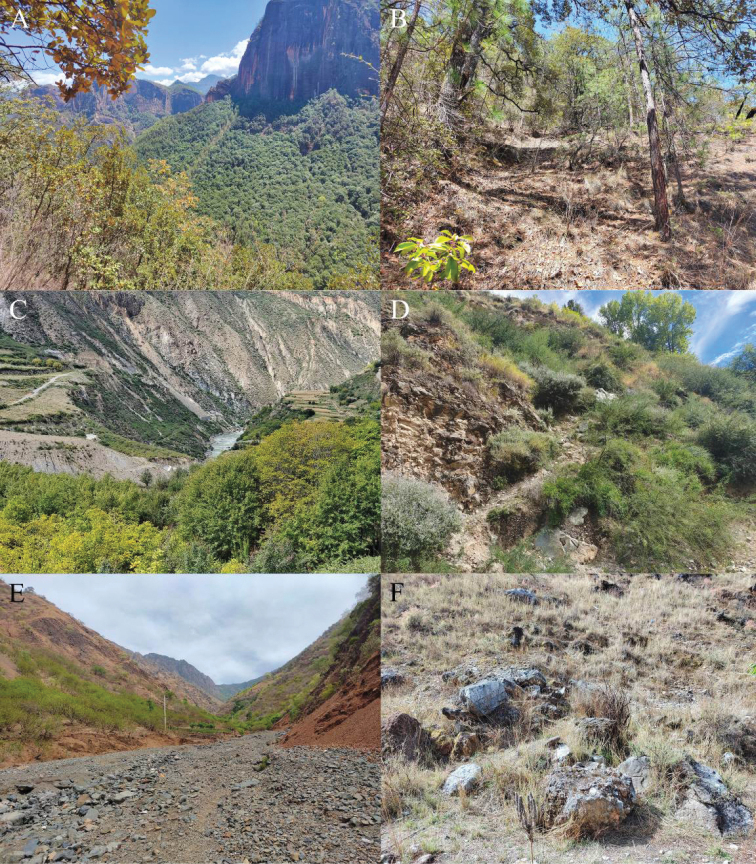
Habitats of the new species **A** distant view of the type locality of *Diplodermalimingense* sp. nov. **B** close view of the type locality of *Diplodermalimingense* sp. nov. **C** distant view of the type locality of *Diplodermashuoquense* sp. nov. **D** close view of the type locality of *Diplodermashuoquense* sp. nov. **E** distant view of the type locality of *Diplodermayongshengense* sp. nov. **F** close view of the type locality of *Diplodermayongshengense* sp. nov.

This species is terrestrial, inhabiting the hot-dry valley. There are a few trees and many rocks at the type locality (Fig. 12E, F). All specimens were collected between 2 and 4 p.m. when they were basking on large rocks, no female or juvenile being found. The population density of this species was relatively high, however, the habitats of this species being seriously threatened by human activities. According to IUCN Criteria, we recommend listing this new species as Near Threatened (NT).

## ﻿Discussion

Species of *Diploderma* can be roughly divided into two ecotypes, one inhabiting mountain forests (i.e. *D.brevicauda*, *D.chapaense*, *D.dymondi*, *D.fasciatum*, *D.menghaiense*, *D.swild*, *D.varcoae*, *D.yunnanense* and *Diplodermalimingense* sp. nov., etc) and the other inhabiting hot-dry river valleys (i.e. *D.aorun*, *D.bowoense*, *D.drukdaypo*, *D.formosgulae*, *D.laeviventre*, *D.vela*, *D.yangi*, *Diplodermashuoquense* sp. nov. and *Diplodermayongshengense* sp. nov. etc). Mountain forest is often distributed in large areas. Unless there are very high mountains or very large rivers through the forest, different populations living in the forest will not be completely separated and there can be gene exchange between them. Therefore, the species inhabiting forests are usually widely distributed and their diversity is usually low. However, in the Hengduan Mountain Region, there are high mountains between the numerous river valleys, in addition, the altitude drop in different sections of the same river is usually large. Different populations living in the valleys are usually separated from each other and it is difficult for them to make gene exchange. Therefore, in contrast to the species inhabiting forests, the species inhabiting river valleys usually have very small distribution ranges and their diversity is usually very high.

Large areas of forest are not easy to be destroyed completely. Even if some parts are destroyed, there will still be many spaces for species to survive. Therefore, the species inhabiting forests are relatively less threatened by humans. On the contrary, if a section of a river valley is destroyed, such as by expansion of townships and agricultural lands, construction of tourist sites, development of highways and construction of hydroelectric plants (Wang et al. 2016, 2019b, 2021a), the endemic species there may become extinct. Therefore, the species inhabiting river valleys are more vulnerable to human threats. We should focus the conservation efforts on the species that inhabit river valleys and strengthen the protection of the ecological environment of the river valleys in the Hengduan Mountain Region. In addition, we should strengthen the survey of this region to clarify the species diversity of this region, so as to better protect the endemic species in this region.

## Supplementary Material

XML Treatment for Diploderma limingense

XML Treatment for Diploderma shuoquense

XML Treatment for Diploderma yongshengense

## Supplementary materials

Supplementary material 1 Uncorrected genetic pairwise distances (*p*-distances) (%) between species based on the mitochondrial ND2 gene sequences Data type Xls file. https://binary.pensoft.net/file/772265This dataset is made available under the Open Database License (http://opendatacommons.org/licenses/odbl/1.0/). The Open Database License (ODbL) is a license agreement intended to allow users to freely share, modify, and use this Dataset while maintaining this same freedom for others, provided that the original source and author(s) are credited.Shuo Liu, Mian Hou, Dingqi Rao, Natalia B. Ananjeva
